# A Novel Role for the Periaqueductal Gray in Consummatory Behavior

**DOI:** 10.3389/fnbeh.2018.00178

**Published:** 2018-08-28

**Authors:** Valerie Lee Tryon, Sheri J. Y. Mizumori

**Affiliations:** ^1^Department of Psychology, University of Washington, Seattle, WA, United States; ^2^Neuroscience Program, University of Washington, Seattle, WA, United States

**Keywords:** periaqueductal gray (PAG), consummatory behavior, appetitive, reward processing, electropysiology

## Abstract

The periaqueductal gray (PAG) has a well-established role in pain processing, autonomic function and behavioral responses to fear. Anatomical work suggests the PAG may mediate food intake and reward processing as it has extensive reciprocal connections within brain circuits that mediate appetitive processes and consummatory behaviors such as prefrontal cortex, hypothalamus, amygdala, parabrachial nucleus (PBN) and ventral tegmental area (Kelley et al., 2005). Therefore, we investigated if the PAG of hungry rats has a functional role in appetitive and consummatory behaviors. To address this, PAG was pharmacologically inactivated during a spatial working memory task with muscimol (0.1–0.3 μg), a GABA_A_ agonist via intracranial infusion. Inactivation of PAG led to reduced intake of food rewards and increased errors on this task. To focus on the specific effects PAG inactivation had on food consumption, PAG was inactivated during two separate food intake tasks in a separate group of rats. Again, PAG inactivation resulted in a significant decrease in food consumption, as well as an increased latency to consume food. We next investigated PAG neural responses to reward encounters. A different group of rats performed the same task used in Experiment 1 while the *in vivo* activity of PAG neurons was recorded. In a subset of PAG neurons, reward encounters elicited phasic excitation. A separate subset of PAG neurons were inhibited during reward encounters. These responses scaled with the size of the reward, with sustained excitation or inhibition in response to large rewards compared to small. Our data also show that separate groups of PAG neurons in awake behaving animals display either increased and decreased neural responses to reward encounters. Additionally, a proportion of neurons were modulated by the animals’ velocity. This study is the first to show that PAG neurons process reward-related information, perhaps to mediate consummatory behaviors related to food consumption.

## Introduction

The periaqueductal gray (PAG) is a structure that has been long-known to play an important role in endogenous analgesia, vocalizations, defensive behaviors and autonomic regulation (Bandler and Depaulis, 1991; Behbehani, 1995). Mounting evidence suggests the PAG is also situated to mediate complex emotional and motivated behaviors through its vast connections throughout the brain (Motta et al., 2017). For example, it is connected to regions important for decision making (e.g., medial prefrontal cortex and amygdala, Beitz, 1982; Rizvi et al., 1991; Rozeske et al., 2018), reward processing and motivation (e.g., the ventral tegmental area, VTA, Omelchenko and Sesack, 2010; Ntamati et al., 2018) and basic homeostatic drives (e.g., the lateral hypothalamus and parabrachial nucleus (PBN), Behbehani et al., 1988; Krout et al., 1998). These and other studies suggest that the PAG may play a functional role in mediating appetitive behaviors but there has not been much investigation of this hypothesis. Thus, the goal of this study was to examine whether the PAG is important in mediating appetitive processes.

Based on its placement within decision-making, reinforcement learning, fear and pain neurocircuitry, the PAG may play a critical role in the integration of threat and other noxious information with homeostatic and basic drives such as hunger to select the most appropriate behavior for a given situation. When faced with competing behavioral options (i.e., to fight or to flee in the face of a threat, to forage or to hide when hungry in times of stress) animals must weigh the relative costs and benefits of each option. This essential process is mediated by a complex circuitry that engages multiple regions of the brain such as prefrontal cortex, hypothalamus, hippocampus, ventral tegmental area, basal ganglia structures and more (Kelley et al., 2005). Indeed, it has been documented that expectancy of a food reward can activate endogenous opioid-mediated analgesia which is thought to be mediated by the PAG (Dum and Herz, 1984; Fields, 2004) and it is hypothesized that reward expectation elicits an analgesic response to allow an animal to ignore noxious stimuli and attend to a rewarding stimulus (Fields, 2007; Leknes and Tracey, 2008).

The PAG sends excitatory and inhibitory projections to both GABAergic and dopaminergic VTA neurons (Omelchenko and Sesack, 2010; Ntamati et al., 2018), providing subcortical glutamatergic input to the VTA (Geisler et al., 2007; Sesack and Grace, 2010). The PAG is also known to directly mediate aspects of reward during drug reinforcement (Brandão, 1993) since mice will self-administer morphine into PAG (David and Cazala, 1994). In addition to being anatomically situated within the reward neurocircuitry, the PAG is also linked to the brain’s core feeding circuit such as the hypothalamus and PBN (Mota-Ortiz et al., 2009; Betley et al., 2013). However, stimulation of PAG-projecting hypothalamic agouti-related protein (AGRP) neurons was not sufficient to induce feeding (Betley et al., 2013) and infusion of morphine into PAG inhibits lateral hypothalamus stimulation-induced feeding (Jenck et al., 1987). Thus, PAG activity may not directly induce feeding behavior, but instead may mediate whether approach behavior is appropriate given the current situation, such as when other behavioral drives (e.g., exogenous threat or maternal drives) compete for an organisms’ resources. Indeed, previous research shows increased Fos expression localized in the lateral PAG during prey-hunting, a food-oriented behavior (Comoli et al., 2003) and infusion of opioid-receptor agonists such as morphine into the lateral PAG can induce rats to switch from maternal behaviors to prey hunting (Sukikara et al., 2006). This effect can be blocked by local infusion of naloxone (Miranda-Paiva et al., 2003). We hypothesized that the PAG could be involved in processing appetitive information to guide adaptive behaviors.

To investigate our hypothesis, we temporarily inactivated the PAG using a GABA agonist while rats ran a maze-based foraging task for rewards. The majority of injections sites were localized to the lateral and dorsolateral columns of the PAG. It was found that rats whose PAG was inactivated made significantly more errors, ate less of the reward and showed decreased preference to first choose large rewards over small rewards. To directly address whether these observed effects were due to altered food consumption abilities, we conducted an additional PAG inactivation study during which we assessed rats’ ability to consume food. We found that rats with an inactivated PAG showed significantly decreased food intake, even while hungry. We then directly measured whether or not PAG neurons encode reward information by recording from PAG neurons in awake, behaving animals that also ran the foraging task. Like the pharmacological inactivation studies, many of the recording sites were focused in the lateral and dorsolateral columns of the PAG. Indeed, we found strongly excited and inhibited PAG neural response to reward. These results, in conjunction with the anatomical evidence, suggest that the PAG may in fact play a role in reward related processing to regulate food intake.

## Materials and Methods

### Subjects

Twenty-three (nine to test PAG inactivation effects on maze performance; seven to test PAG inactivation effects on food intake; seven for PAG neural recordings); male Long–Evans rats (340–460 g; Simonson Laboratories) were housed individually in Plexiglas cages. The rats were maintained on a 12 h light/dark cycle (lights on at 7:00 A.M.) and all behavioral experiments were performed during the light phase of the cycle. Each rat was allowed access to water *ad libitum* and food-deprived to 85% of its *ad libitum* feeding weight. Rats were handled and weighed daily for the duration of the experiments (30–120 days). All animal care and use were conducted in accordance with University of Washington’s Institutional Animal Care and Use Committee guidelines. The protocol was approved by University of Washington’s Institutional Animal Care and Use Committee.

### Differential-Reward, Spatial Memory Task

**Figure 1:**
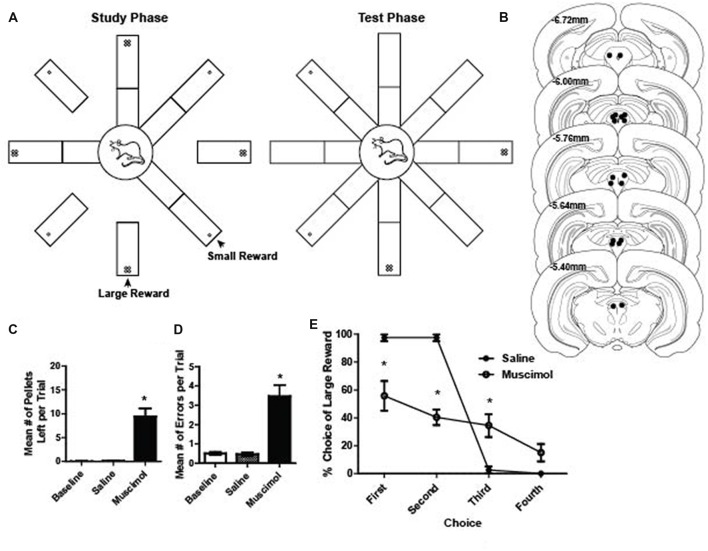
**(A)** Schematic illustration of the radial arm maze task. During the study phase, one arm of the maze was raised at a time until four arms were visited by the rat. Large rewards (four sugar pellets) were placed on every other arm of the maze. During the test phase, all eight arms of the radial maze were raised and rats were free to visit any arm of their choice to collect the rewards from the remaining four arms. **(B)** Reconstruction of bilateral cannulae tip placement relative to Bregma (*N* = 9). **(C)** Mean number of pellets per trial that were left on the maze during baseline, saline and muscimol trials. A total of 20 pellets were available per trial. There were significantly more pellets left during muscimol trials compared to baseline and saline (dependent *t* test, saline vs. muscimol normalized to baseline pellets; *t*_(1,17)_ = 5.59, *p* < 0.001). **(D)** Mean number of errors per trial during baseline, saline and muscimol conditions. There were significantly more errors on muscimol trials compared to baseline and saline (dependent *t* test, saline vs. muscimol normalized to baseline errors, *t*_(1,17)_ = 5.46, *p* < 0.001). **(E)** Preference for visiting the large reward arms first during the test phase of a trial for saline- and muscimol-treated rats. Muscimol-treated rats showed a significantly reduced preference to collect large rewards on the first and second free choice when compared to saline-treated rats (two-way repeated measures ANOVA, main effect of treatment: *F*_(1,7)_ = 23.19, *p* < 0.001; main effect of choice order: *F*_(3,21)_ = 61.47, *p* < 0.001; significant interaction between treatment and choice order: *F*_(3,21)_ = 23.44, *p* < 0.001). All error bars represent ±SEM. *p ≤ 0.05.

Detailed information of the apparatus and training procedures can be found in previous studies (Pratt and Mizumori, 2001; Puryear et al., 2010; Norton et al., 2011; Jo et al., 2013). Briefly, rats were familiarized with an elevated eight-arm maze (79 cm from the floor; Figure 1A) and allowed to freely forage for sugar pellets (45 mg sucrose tablets, TestDiet) scattered on black Plexiglas arms (58 × 5.5 cm each) that radiated from a circular central platform (19.5 cm in diameter) for 3 days. Each maze arm was hinged such that its proximal end to the central platform could be raised and lowered by remote control from an adjacent room. The maze was surrounded by black curtains on which hung several visual cues. Once the rat consistently moved about and consumed rewards on the maze, the training for a spatial memory task started. While a rat was constrained to the central platform by lowering all maze arms (Figure 1A), food cups located at the end of the maze arms were baited with either a large (four pellets) or small (one pellet) reward of 45 mg sugar pellets on alternating arms (e.g., large rewards on even-numbered arms and small rewards on odd-numbered arms; counter-balanced across rats). Subsequent training trials consisted of a study and a test phase. During the study phase of each trial, four of the eight arms (two large reward and two small-reward arms) were randomly selected and presented individually. After presentation of the fourth arm, the test phase began upon making all maze arms accessible at once. The rat was required to collect the remaining rewards from the four arms not presented during the study phase. Revisits (i.e., when animals went at least halfway down a maze arm) to previously visited arms within a trial were defined as errors. When the animal returned to the central platform after visiting all eight arms, it was confined to the platform for an inter-trial interval (ITI) of 60 s. Meanwhile, all food cups were baited again and 10 trials, separated into two blocks of five trials, were given per day. The locations of differentially rewarded arms were held constant for each rat throughout training. After rats made an average of one or fewer errors per trial on a training day, rats underwent a surgical procedure for the implantation of recording electrodes or cannulae. Rats in the PAG inactivation experiments were tested a total of four times each: two saline sessions and two muscimol sessions. Rats undergoing saline-first or muscimol-first was counterbalanced across rats.

For the neural recording study, the first block of five trials served as the baseline trials and was the same as described above. For the second block of five trials, one of three manipulations was administered: reward-switch, omission, or no manipulation. For the reward switch manipulation, the learned locations of the small and large rewards were switched. For the omission manipulation, two of the four arms visited during the study phase (one large and one small reward arm) had omitted rewards.

### Food Intake Tests

For the first food intake test, rats were food restricted to 80%–85% of their free-feeding weight and fasted for 24 h prior to the food intake test. They had *ad libitum* access to water at all times. Prior to the testing days, rats were habituated to the arena for 2 days. The first day, rats were placed in the arena for 20 min each. On the second day, each rat received a saline infusion, to habituate them to the infusion process, and then were placed in the arena for 20 min. During the test, rats were randomly placed in one of the four corners of the food intake arena (40 cm × 40 cm × 40 cm tall). In one of the corners was a plastic food cup filled with 1-g regular rat chow pellets (Test diets filled cups that were affixed to the floor of the arena with Velcro). Once rats were placed in the arena they had free access to the rat chow. After 10 min, the food cup was quickly replaced with a full one and weighed. This switching/weighing occurred again after 20, 30, 60, 90 and finally after 120 min. Rats were continuously monitored and tracked using ANY-maze software and a camera mounted above the arena. After 2 h had expired, the rat was removed from the arena and returned to its home cage. Any food particles left in the arena were collected and weighed. The arena and food cup were cleaned with Virkon (1%) solution between rats. Each rat was tested twice, and performed the food intake test for one saline infusion session and one muscimol infusion session. The order of saline vs. muscimol injection first was counterbalanced across rats.

The second food intake test quantified the latency to consume a palatable reward. As with the first food intake test, rats were maintained at 80%–85% free feeding weight and fasted 24 h prior to the sucrose test. Rats were tested in the same arena as the first food intake test. In the center of the arena a food cup was affixed to the arena floor with Velcro. Rats were placed in the arena and habituated for 2 min prior to the beginning of the first trial. To begin a trial, four 45 mg sugar pellets were placed in the food cup. The latency to consume all four sugar pellets was recorded. Once the rat consumed all pellets, the ITI of 60 s began. Rats were given 120 s to consume the four pellets before they were removed from the maze. At that time, the next ITI began. Fresh pellets were given at the beginning of each trial even if the rat failed to consume them in the previous trial. A session consisted of 20 trials and lasted 15–45 min depending on duration to consume sucrose pellets. Each rat was tested twice for the latency to consume sucrose test, and performed one saline infusion session and one muscimol infusion session. The order of saline vs. muscimol injection first was counterbalanced across rats.

### Electrode, Cannula and Surgical Procedures

Recording tetrodes were constructed from 20 μm lacquer-coated tungsten wires (California Fine Wire, Grover Beach, CA, USA) and mounted on an array of three or four independently adjustable custom made microdrives (two or three tetrodes per microdrive). Tetrode tips were gold-plated to reduce impedance to 0.1–0.4 MΩ (tested at 1 kHz). The guide cannulae (Plastics One, Roanoke, VA, USA) were composed of 26-gauge stainless steel tubes cut to custom lengths (see below) whereas the injection cannulae were 33 gauge, cut to extend 1.0 mm below the guide cannulae. Each rat was placed in an induction chamber and deeply anesthetized under isoflurane (4% mix with oxygen at a flow rate of 1 L/min). Under deep anesthesia, the animal was placed in a stereotaxic instrument (David Kopf Instruments, Tujunga, CA, USA) and anesthesia was maintained throughout surgery by isoflurane (1%–2.5%) delivered via a nosecone. The skull was exposed and adjusted to place bregma and lambda on the same horizontal plane. After small burr holes were drilled, two 25-gauge cannulae or the microdrive were bilaterally implanted into the PAG (6.0 mm posterior, 0.5 mm lateral and 6.0 mm ventral to bregma). The cannulae bilaterally targeted the PAG while the microdrive array was unilaterally implanted. Cannulae and microdrive arrays were secured in place with anchoring screws and dental cement. A 33-gauge dummy cannula was inserted into each guide to prevent clogging. Rats were allowed to recover for 7 days, during which they were weighed and handled daily.

### Intracranial Microinjection

Muscimol (a GABA_A_ agonist; 1 μg/μl dissolved in 0.9% saline) was used to temporarily inactivate the PAG. Microinjection procedures were performed as previously described (Jo et al., 2013). Briefly, a 33-gauge injection cannula extending 1 mm below the tip of the guide cannula was connected to a 10 μl syringe (Hamilton) via polyethylene tubing (PE 20). Prior to tests days for the maze and food intake tests, each rat received an injection of 0.9% saline to habituate them to the injection protocol. On test days, 0.1–0.3 μl/side of either muscimol or saline was injected bilaterally at a rate of 12 μl/h using an infusion pump (KD Scientific). The injection cannulae were left in place for an additional 1 min to allow diffusion of the drugs from the injection tip. Rats were then returned to their home cages, and were closely observed for 10–20 min before they were placed on the maze or in the food intake test box. Drug was injected into the PAG before the second block of five trials on the maze. Each rat experienced both muscimol and saline injection days, and saline or muscimol was injected in random orders across rats.

### Single-Unit Recording and Postsurgical Procedures

After a week of recovery, rats were returned to a food-restricted diet and spontaneous neural activity in the PAG was monitored as follows: the electronic interface board (Neuralynx) of the microdrives was connected to preamplifiers, and the outputs were transferred to a Cheetah data acquisition system (Neuralynx). Signals were filtered between 0.6 kHz and 6 kHz, and digitized at 16 kHz. Neuronal spikes were recorded for 2 ms after the voltage deflection exceeded a predetermined threshold at 500–7,000× amplification. If no units were encountered, tetrodes were lowered in 40 μm increments to target new units. A video camera mounted on the ceiling tracked infrared LED signals attached to the preamplifier and subsequent position data were relayed to the acquisition system. Once clearly isolated and stable units were found, recording on the maze began. Experimental sessions continued until tetrodes passed through the PAG based on the distance traveled from the brain surface.

### Histology

After the completion of all recording sessions, cannula positions and tetrode locations were verified. Rats were deeply anesthetized under 4% isoflurane. For neural recordings, the final position of each tetrode was marked by passing a 15 A current through a subset of the tetrode tips for 15 s. Then, the animals were given an overdose of sodium pentobarbital and transcardially perfused with 0.9% saline and a 10% formaldehyde solution. Brains were stored in a 10% formalin–30% sucrose solution at 4°C for 72 h. The brains were frozen, and then cut in coronal sections (45 μm) on a freezing sliding microtome. The sections were then mounted on gelatin-coated slides, stained with cresyl violet, and examined under light microscopy. Only cells verified to be recorded in PAG were included in the data analysis and animals with both cannulae in the PAG were included in the behavioral analysis in the inactivation studies.

### Data Analysis

PAG single units were isolated using an Offline Sorter (Plexon). Various waveform features, such as the relative peak, valley, width and principle component, were compared across multiple units simultaneously recorded from the four wires of a tetrode. Only units showing good recording stability across blocks were included. Further analysis of the sorted units was performed with custom Matlab software (Mathworks, Natick, MA, USA). To examine the reward-related responses of PAG neurons, peri-event time histograms (PETHs) were constructed at 4.0 s around the time of all reward acquisition-triggered events. A bin size of 50 ms was used for all PETHs.

### Statistical Analysis

Statistical analyses were performed using SPSS 19.0, Graphpad Prism 6.0, or custom Matlab software (Mathworks, Natick, MA, USA). Drug effects on behavioral performance on the radial maze were analyzed with dependent *t* tests (a rat’s drug performance was compared to its own saline performance, normalized to baseline performance) for pellet consumption and errors. Preference for large reward was assessed with a multifactorial ANOVA (repeated measures for choice and drug group as another within subjects factor) followed by Bonferroni’s *post hoc* pairwise comparisons. Drug effects on the food intake tests were analyzed with dependent *t* tests (rats’ food intake, movement variables, or latency to consume sucrose was compared for saline and muscimol). Cumulative food consumed was assessed with repeated measures ANOVA.

Reward-related PAG cells were identified based on phasic responses to reward acquisition (Martig and Mizumori, 2011; Jo et al., 2013). A PAG neuron was considered reward responsive if it had significantly elevated or decreased firing during the 500 ms after obtaining a large reward compared to the neuron’s baseline firing rate as measured by a Wilcoxon signed rank test. Based on these results, neurons were grouped into reward-inhibited, reward-excited, or not reward modulated. Subsequently, the firing rate patterns of those same cells were assessed relative to encounters with small rewards to determine whether there were differences in firing depending on reward magnitudes. Neural responses across blocks (for reward switch conditions) or reward encounter type (large, small or no reward) were analyzed with a two-way repeated-measures ANOVA, followed by Bonferroni’s *post hoc* pairwise comparisons. Two-way ANOVA (block as within subjects’ factor, manipulation type as between subjects’ factor) was used to assess neural responses for the reward switch conditions. These tests were performed separately for each reward response group, i.e., excited, inhibited, or not modulated by reward. Pearson’s correlation tests were used to assess relationships between firing rate and velocity. Two-tailed *p* values of ≤0.05 were considered statistically significant, unless otherwise noted. All data are expressed as mean ± SEM, unless otherwise noted.

## Results

### PAG Inactivation Effects on Radial Maze Performance

Our initial investigation into whether or not PAG plays a role in reward processing was assessed by temporarily inactivating the PAG with muscimol, a GABA_A_ receptor agonist, while rats ran on a radial eight arm maze to collect rewards (sugar pellets; Figures 1A,B). GABA_A_ receptors are present throughout the entire PAG matter of the rat (Griffiths and Lovick, 2005). Using the same task, previous research has shown that inactivating the VTA resulted in compromised working memory performance (Martig et al., 2009; Martig and Mizumori, 2011). We hypothesized that if PAG is necessary for processing information about the presence of rewarding stimuli, the consumption of palatable rewards would be impaired. Additionally, because of its anatomical connection with regions important for decision making such as ventral tegmental area and prefrontal cortex (Floyd et al., 2000; Omelchenko and Sesack, 2010; Ntamati et al., 2018), we hypothesized that we would also see compromised performance when inactivating PAG because the appropriate computations necessary for optimal performance based on working memory and incentive salience (Berridge and Robinson, 1998) would be compromised. To assess this, we compared the number of errors made, the number of pellets eaten, and their preference for a large or small reward in baseline, vehicle and inactivation conditions. It was found that when compared to control sessions, muscimol-treated rats made significantly more errors (dependent *t* test, saline vs. muscimol normalized to baseline errors, *t*_(1,17)_ = 5.46, *p* < 0.001; Figure 1D) and they ate less of the reward (dependent *t* test, saline vs. muscimol normalized to baseline pellets; *t*_(1,17)_ = 5.59, *p* < 0.001; Figure 1C). When comparing the preference to retrieve large rewards during the test phase of each trial, it was found that muscimol-treated rats showed a significantly decreased preference to choose large rewards first (two-way repeated measures ANOVA, main effect of treatment: *F*_(1,7)_ = 23.19, *p* < 0.001; main effect of choice order: *F*_(3,21)_ = 61.47, *p* < 0.001; significant interaction between treatment and choice order: *F*_(3,21)_ = 23.44, *p* < 0.001; Figure 1E). It was found that when comparing the first four choices during the test phase individually, muscimol-treated rats showed a consistently lower overall preference for large rewards except for on the fourth choice (Bonferroni *post hoc* comparisons, all *p* < 0.05 except fourth choice; Figure 1E). These results, taken together with the anatomical evidence, suggest that the PAG may in fact play a role in reward related processing.

### PAG Inactivation Effects on Food Intake

**Figure 2:**
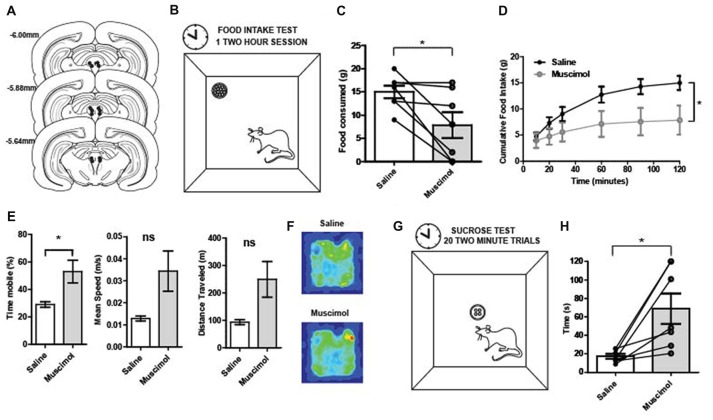
**(A)** Reconstruction of bilateral cannulae tip placement (*N* = 7) that targeted the periaqueductal gray (PAG). **(B)** Food intake test: during the 2-h test, rats were placed in the arena (40 × 40 × 40 cm). A food cup was filled with 1 g chow pellets. Food was weighed at 20, 30, 60, 90 and 120 min. **(C)** Food in take test results: following temporary inactivation of the PAG, the total amount of food consumed over 2 h was significantly decreased compared to vehicle treated rats (*t*_(6)_ = 2.95, *p* = 0.01). **(D)** PAG inactivation produced changes in cumulative food intake over time. There was a main effect of time (two-way repeated measures ANOVA, *F*_(5,30)_ = 21.23, *p* < 0.01), no main effect of treatment group (*F*_(1,6)_ = 4.37, *p* = 0.08), but a significant interaction between time and treatment group (*F*_(5,30)_ = 22.24, *p* < 0.01). The two treatment groups were significantly different (*p* < 0.05) towards the end of the testing period, at the 90 and 120-min time points. **(E)** Movement data collected during the 2-h food intake test revealed that reduced consumption was not due to less activity. Muscimol-treated rats showed increased locomotion relative to saline on measures in terms of total time mobile (*t*_(6)_ = 2.71, *p* = 0.04). **(F)** Average occupancy data over the 2 h food intake test for each group of rats. The total time in various arena locations were similar between saline- and muscimol-treated rats. **(G)** Latency to consume sucrose test: four 45 mg sugar pellets were placed in the food cup. The latency to consume all four sugar pellets was recorded. Rats were given 120 s to consume the four pellets before they were taken away and the inter-trial interval (ITI) began. A session consisted of 20 trials. **(H)** Latency to consume sucrose after PAG inactivation: Individual points represent rats’ mean latency to consume sucrose pellets over 20 trials. The time it took muscimol-treated rats to consume four 45 mg sugar pellets was significantly increased relative to vehicle treated trials (*t*_(6)_ = 3.37, *p* = 0.01). All error bars represent ±SEM. *p ≤ 0.05; ns, not significant.

While intriguing, the radial maze inactivation study data do not directly support the conclusion that PAG is necessary for reward consumption *per se* as the requirement of animals to complete a complex spatial working memory task introduces multiple alternative explanations for the results observed. To explicitly test the effect of PAG inactivation on food consumption, an additional inactivation study was performed with a separate group of rats (Figure 2B). Histological reconstructions show that cannula tips were not in exactly the same location of the PAG for each rat (Figure 2A). Previous research has shown functional differences for each column of the PAG (Carrive, 1993; Bandler and Shipley, 1994). Thus, rats were grouped by column of the PAG that was targeted. Since no significant behavioral differences were observed (Supplementary Figure S1), animals’ data were grouped together for subsequent analyses.

PAG inactivation significantly reduced the total amount of food consumed over the 2-h food intake test compared to control sessions (*t*_(6)_ = 2.95, *p* = 0.01; Figure 2C). Regarding cumulative food intake over time, there was a main effect of time (two-way repeated measures ANOVA, *F*_(5,30)_ = 21.23, *p* < 0.001), no main effect of treatment group (*F*_(1,6)_ = 4.37, *p* = 0.08), but a significant interaction between time and treatment group (*F*_(5,30)_ = 22.24, *p* < 0.001; Figure 2D). While the PAG-inactivated animals were still able to consume food, the total amount of food consumed was significantly diminished compared to vehicle treatment especially at the end of the test period. It is possible that the observed effect was due to a general motor impairment. Thus, movement data collected during the 2-h food intake test were analyzed to see if muscimol-treated rats had significant impairments in general locomotor behavior. However, muscimol-treated rats were not impaired in speed or total distance traveled. Rather they showed increased locomotion relative to saline on measures in terms of total time mobile (*t*_(6)_ = 2.71, *p* = 0.04; Figures 2E,F).

The latency to consume sucrose test revealed a similar impairment in food consumption for PAG inactivated rats (Figure 2G). The average time it took muscimol-treated rats to consume four 45 mg sugar pellets was significantly increased relative to vehicle treated trials (*t*_(6)_ = 3.37, *p* = 0.01; Figure 2H). The results provide evidence that PAG is needed for normal consummatory behaviors for both regular food and palatable rewards.

### PAG Neural Activity During Maze Performance

**Figure 3:**
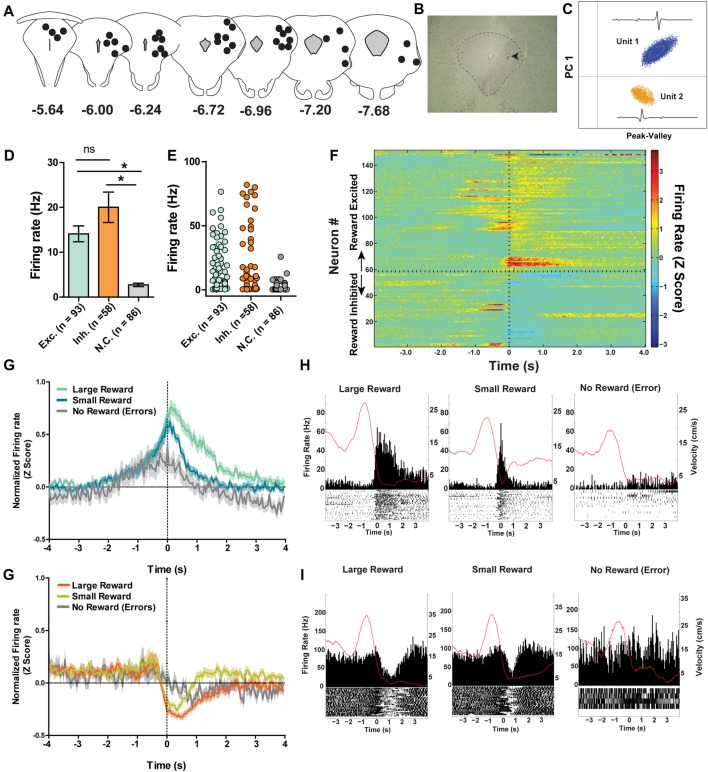
**(A)** Reconstruction of terminal tetrode locations relative to Bregma (mm). Black dots represent the location of the electrolytic lesion that marked the tetrode location. **(B)** Example electrolytic lesion in the PAG. **(C)** Illustration of signals from two simultaneously recorded PAG neurons. **(D)** A total of 237 individual neurons from seven rats were recorded from the PAG during spatial working memory task performance (see Figure 1A). Individual neurons were sorted into one of three groups: reward excited, reward inhibited, or not significantly reward modulated (Wilcoxon signed rank test, see “Materials and Methods” section). Reward modulated neurons had significantly higher firing rates than non-reward modulated cells (one-way ANOVA, *F*_(2,24)_ = 21.08, *p* < 0.0001; Bonferroni’s *post hoc* comparisons to compare each pair, *indicates *p* < 0.05). Exh. = Reward Excited, Inh. = Reward Inhibited, N.C. = not correlated to reward. Error bars represent ±SEM. **(E)** Each dot represents the mean firing rate of an individual neuron from each group. **(F)** Heatmap of normalized firing rates of all reward responsive neurons. Time 0 indicates onset of large reward encounters. Dotted line separates the reward excited and reward inhibited groups. As observed, some reward responsive neurons showed changes in firing patterns before reward onset began while others increased or decreased firing after reward encounters. **(G)** Normalized firing rates of reward excited neurons in response to large, small and no rewards. Time 0 indicates onset of reward encounters for large and small rewards or expected reward location for error trials. The solid line indicates mean firing rate within 50 ms time bins; the shaded region around each line represents ±SEM. The magnitude of reward responses scaled with reward magnitude (see “Results” section). **(H)** Peri-event time histograms (PETHs) of a reward excited neurons’ responses to large, small, and no rewards. Left *Y* axis represent the mean firing rate (Hz) over all events; right *Y* axis is mean velocity (cm/s), represented by the red line. **(I)** Normalized firing rates of reward inhibited neurons in response to large, small, and no rewards. **(J)** PETHs of a reward-inhibited neurons’ responses to large, small and no rewards. *p ≤ 0.05; ns, not significant.

A total of 237 individual neurons were recorded across the different columns of the PAG from seven rats as they ran on a spatial working memory maze task (Figures 3A–C). Of particular interest was whether or not these neurons would show distinct neural correlates with reward encounters. Thus, neurons were first sorted into one of three groups based on their phasic responses to reward encounters: reward excited (*n* = 93), reward inhibited (*n* = 58), or not significantly affected by reward (*n* = 86; Figures 3D–F; see “Materials and Methods” section). Then, whether or not reward magnitude had any effect on these phasic reward-induced responses was assessed by comparing each neuron’s firing rate during the time just before (two 250 ms epochs −500 ms to 0 ms) and after (0 ms to 500 ms) reward encounters for large rewards, small rewards and no rewards. For the reward excited neurons, there was a significant main effect of reward size (Two-way repeated measures ANOVA, *F*_(2,180)_ = 21.23, *p* < 0.001) time (*F*_(3,270)_ = 5.23, *p* = 0.002), and a significant interaction between reward and time (*F*_(6,540)_ = 11.17, *p* < 0.001; Figures 3G,H). *Post hoc* pairwise comparisons (comparing all combinations of pairs, i.e., large reward with small, small with no reward, and large with no reward with a Bonferroni adjustment for multiple comparisons) show that there were significant differences comparing all levels of reward size (*p* < 0.001 for all comparisons). The firing rate of reward excited neurons was highest in response to large rewards compared to small rewards and no rewards. Small rewards elicited a higher firing rate than no reward. *Post hoc* pairwise comparisons for the significant main effect of time (comparing all combinations of pairs, i.e., −500 ms to −250 ms with −250 ms to 0 ms and so on) revealed that only the time period furthest from reward encounters (−500 ms to −250 ms) was significantly different from firing rates centered around reward consumption (−250 ms to 0 ms; *p* = 0.01; 0 ms to 250 ms; *p* = 0.01). In other words, during the time directly preceding and directly after the reward encounter, reward excited neurons exhibited an increased firing rate preceding a reward encounter and these peri-reward time periods were not significantly different 250–500 ms after reward encounters, showing that this excitatory response was sustained. Because this excitation began directly prior to reward encounters likely reflects anticipation or sensory information, indicating reward as imminent. This analysis shows that for neurons classified as reward excited, the size of reward and time from reward had a significant influence on PAG neurons’ firing rate. This suggests that these neurons are not only coding the presence of food, but other sensory factors that are related to the food consumption period.

The same analysis was repeated for the reward inhibited neurons, which revealed a significant main effect of reward magnitude (Two-way repeated measures ANOVA, *F*_(2,108)_ = 17.47, *p* < 0.001) time (*F*_(3,162)_ = 15.11, *p* < 0.001), and a significant interaction between reward and time (*F*_(6,324)_ = 6.82, *p* < 0.001; Figures 3I,J). *Post hoc* pairwise comparisons showed that firing rates in response to large and small rewards were not significantly different (*p* = 0.06). Although not significant, the data do show that the inhibition response appears to be sustained for longer with large reward compared to small (Figures 3I,J). Furthermore, neurons’ responses to no reward were significantly reduced, as the magnitude of inhibition was not as great, compared to the inhibition observed for neural responses to both large reward encounters (*p* < 0.001) and small reward encounters (*p* < 0.001). *Post hoc* pairwise comparisons for the significant main effect of time revealed that all pairwise comparisons for the different time points were significantly different (*p* < 0.05) except for when comparing the latest time periods (*p* = 0.67). In other words, the firing rate of reward inhibited neurons was significantly reduced during reward encounters compared to pre-reward encounters, and the degree of firing rate inhibition was maintained for at least 500 ms post-reward encounter. Interestingly, the neurons exhibited reduced firing rate in the period just prior to reward encounters compared to the earliest period which could reflect anticipation or expectation. This analysis shows that for reward inhibited neurons, time from reward encounter and presence of reward (rather than reward magnitude) is also encoded in these neurons’ firing rates.

It is important to note that while PAG neurons could be functionally subdivided into three separate groups based on their time-locked responses to rewarding stimuli, each subgroup of neurons displayed heterogeneity in terms of the details of their responses. For example, within the reward excited neuron group, some neurons showed a sustained phasic excitation to rewards that was initiated before reward encounters, whereas other neurons only showed reward-correlated excitation once the reward location was reached. While there was a lot of variance in the firing rate across individual neurons, the mean rate of the neurons that did not respond to reward encounters was significantly lower than that of the reward correlated neurons (one-way ANOVA, *F*_(2,235)_ = 21.08, *p* < 0.001) while the firing rates of excited and inhibited neurons were not significantly different from each other (Figures 3D,E). It could be that the different subgroups of reward-responsive neurons are comprised of distinct neuronal population (e.g., interneurons vs. projection neurons), but future work is needed to delineate whether or not this is the case.

**Figure 4:**
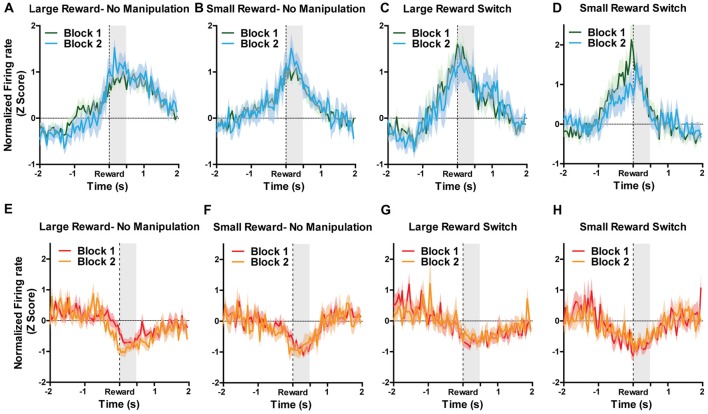
For all figures, the *Y* axis represents the normalized firing rate during a 4 s window centered on reward encounters. *X* axis represents time. Block 1 are the first five trials during which sizes of rewards were always located as expected. During Block 2, rewards could remain in their expected location (no manipulation) or could be switch (reward switch condition). For “large reward switch,” during Block 2, rats received a large reward when they were expecting a small reward. For “small reward switch,” during Block 2, rats received a small reward when they were expecting a small reward. A two-way ANOVA for manipulation type (none or reward switch) and Block (1 and 2) were performed separately for small and large rewards on the normalized firing rates during the first 500 ms after reward encounters (shaded region). Graphs represent normalized responses of **(A)** Reward excited neurons to large rewards when there was no manipulation in Block 2. **(B)** Reward excited neurons to small rewards when there was no manipulation in Block 2. **(C)** Reward excited neurons to large rewards when reward locations were switched in Block 2. **(D)** reward excited neurons to small rewards when reward locations were switched in Block 2. **(E)** Reward inhibited neurons to large rewards when there was no manipulation in Block 2. **(F)** Reward inhibited neurons to small rewards when there was no manipulation in Block 2. **(G)** N reward inhibited neurons to large rewards when reward locations were switched in Block 2. **(H)** Reward inhibited neurons to small rewards when reward locations were switched in Block 2. Block order and manipulation did not significantly affect firing rates during reward encounters for small or large rewards for either reward excited or reward inhibited neurons (all *p’s* > 0.05; see “Results” section).

Because reward-encoding PAG neurons displayed anticipatory activity prior to food consumption, we investigated whether expectation of rewards, and more specifically, violation of those expectations, is reflected in PAG neural responses. To examine this, we analyzed data taken from “reward switch” sessions during which the well-learned location of large and small rewards was switched for Block two trial. Because rats were very familiar with the placement of these rewards (as evidenced by their preference to visit large rewards first), it follows that they had a reward size expectation when making particular choices and that encountering smaller-than or larger-than-expected rewards would violate their expectations, producing reward prediction error signals. Previous research has shown that VTA neurons significantly increase firing to larger than expected rewards when using this same task design (Puryear et al., 2010; Jo et al., 2013). To test if violation of expectation of reward size was reflected in PAG neurons reward responses, a two-way ANOVA for manipulation type (none or reward switch) and Block (1 and 2) were performed separately for small and large rewards on the normalized firing rates during the first 500 ms after reward encounters (Figure 4). For reward excited neurons, the responses to large rewards were not significantly affected by order (aka block; *F*_(1,93)_ = 0.16, *p* = 0.69) or manipulation (*F*_(1,93)_ = 0.94, *p* = 0.34; Figures 4A,C). The results were similar for responses to small rewards, as block (*F*_(1,100)_ = 0.32, *p* = 0.89) and manipulation (*F*_(1,100)_ = 0.37, *p* = 0.54) did not significantly affect normalized firing rate (Figures 4B,D). For reward inhibited neurons, similar results were observed for both large reward (no significant effects of block (*F*_(1,57)_ = 0.11, *p* = 0.74) or manipulation (*F*_(1,57)_ = 1.38, *p* = 0.25; Figures 4E,G) and small rewards (block: (*F*_(1,49)_ = 0.01, *p* = 0.91), manipulation: (*F*_(1,49)_ = 0.00, *p* = 0.99; Figures 4F,H). These data support the conclusion that PAG neurons’ response to reward encounters is not affected by expectation of reward size, but rather the magnitude of change in firing rate is governed by the current magnitude of reward.

**Figure 5:**
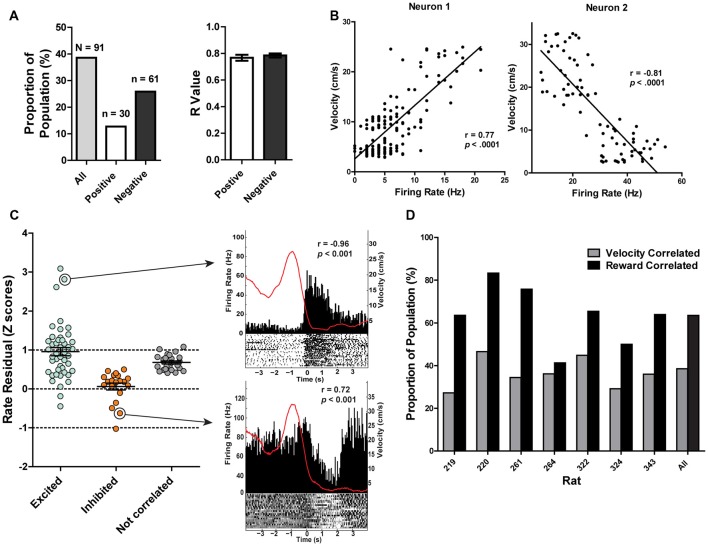
**(A)** 38.40% of recorded PAG neurons were significantly correlated with velocity (Pearson correlation, *p* ≤ 0.05). Of those, 12.66% were positively correlated while 25.73% were negatively correlated. The mean *R* values of positively- and negatively-correlated neurons were similar. **(B)** Example of relationship between firing rate and velocity for a positively-correlated neuron (left) and negatively-correlated neuron (right). *Y-axis* represents velocity (cm/s) while the *x-axis* represents the corresponding firing rate (Hz) from the same time bin. **(C)** Normalized firing rate residuals of velocity-correlated neurons from what would be predicted by velocity during the period when an animal encountered rewards. Neurons are separated into groups based on their relationships to rewards. Residuals close to 0 indicate firing rate does not deviate from velocity-predicted values. The right panel represents two velocity correlated neurons that did not have residuals close to 0. PETHs represent each neuron’s responses to large rewards. Left *Y* axis represent the mean firing rate (Hz) over all events; right *Y* axis is mean velocity (cm/s), represented by the red line. Time 0 indicates onset of large reward encounters. *p* and *r* values represent that neuron’s relationship to velocity. **(D)** Proportion of the population of recorded neurons that are correlated to velocity or reward separated by rat. Note that a neuron could be represented in either group. All rats had more reward correlated than velocity correlated neurons, although the exact proportion did vary from rat to rat.

Dual encoding of both movement and reward has been previously found in other midbrain regions such as the VTA (Puryear et al., 2010) and LDTg (Redila et al., 2015), and thus we examined movement-related correlations in PAG neurons. It was found that the firing rates of 38% (*n* = 91) of recorded neurons were significantly correlated with the rats’ velocity of movement across the maze (*p* ≤ 0.05; Figures 5A,B). The majority of velocity correlated neurons were negatively correlated (negatively correlated: 67%, *n* = 61, mean *R* = −0.78 ± 0.2; positively correlated: 33%, *n* = 30, mean *R* = 0.77 ± 0.02; Figure 5A). Of those significantly correlated to velocity, 62 (69%) were also significantly correlated with reward. To parse out and control for velocity’s contribution to changes in firing rate separate from reward consumption, we fit a linear equation of each neuron with velocity as the predictor during a window where the rats were not getting rewarded. Then, we calculated the difference between the expected firing rate based solely on the relationship between firing rate and velocity already calculated and the observed firing rate during the period where the rat was getting rewarded (Figure 5C). We termed this difference in expected and observed firing rate the rate residual. Residuals close to 0 indicate firing rate does not deviate from velocity-predicted values and that reward consumption does not have an additional effect on firing rate. Conversely, residuals not close to 0 indicate that these neurons are correlated to both movement and reward consumption. Interestingly, some velocity correlated neurons did not have residuals close to 0, suggesting that in these neurons there is conjunctive encoding of both movement and reward information. We also separated correlated neurons by rat and found that all rats had more reward correlated than velocity correlated neurons, although the exact proportion did vary from rat to rat (Figure 5D).

**Figure 6:**
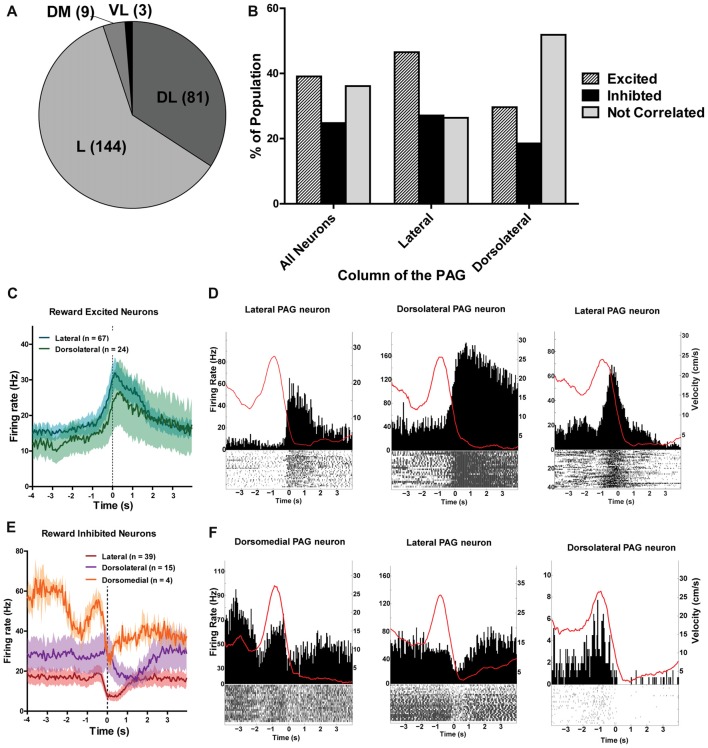
**(A)** Number of PAG neurons recorded from each column of the PAG. The majority of neurons were recorded from the lateral and dorsolateral column. L, lateral; DL, dorsolateral; DM, dorsomedial; VL, ventrolateral. **(B)** Proportion of population of neurons from each column that were reward excited, reward inhibited, or not correlated to reward. The lateral column had more reward excited neurons (*χ^2^*_(2)_ = 10.56; *p* = 0.005) and the dorsolateral column had more non-reward correlated neurons (*χ*^2^_(2)_ = 13.86; *p* = 0.001) than expected based on the responses of all neurons recorded. **(C)** Mean firing rates of reward excited neurons in response to large rewards from the lateral and dorsolateral column. The ventrolateral and dorsomedial column were not represented here as there was only one or two neurons from each column. Time 0 indicates onset of reward encounters. The solid line indicates mean firing rate within 50 ms time bins; the shaded region around each line represents ±SEM. **(D)** PETHs of reward-excited neuron’s responses to large rewards from the lateral (left), dorsolateral (middle), and lateral column (right). Left *Y* axis represent the mean firing rate (Hz) over all events; right *Y* axis is mean velocity (cm/s), represented by the red line. Time 0 indicates onset of large reward encounters. **(E)** Mean firing rates of reward inhibited neurons in response to large rewards by column. Time 0 indicates onset of reward encounters for large rewards. **(F)** PETHs of reward-inhibited neuron’s responses to large rewards from the dorsomedial (left), lateral (middle), and dorsolateral column (right).

The different columns of the PAG have been functionally separated based on their distinct contributions to analgesia, defensive behaviors and autonomic regulation (Carrive, 1993; Bandler and Shipley, 1994). Thus, it stands to reason that the different columns of the PAG may process reward encounters differently. To investigate this, all recorded neurons were separated into different columnar groups: dorsomedial, dorsolateral, lateral and ventrolateral (Figure 6; see also Figure 3A). Unfortunately, not all of the columns were sampled equally and the majority of the neurons were recorded from the lateral (144/237; 60.76%) and dorsolateral columns (82/237; 34.6%; Figure 6A). Nevertheless, we found that the lateral column had more reward excited neurons (*χ^2^*_(2)_ = 10.56; *p* = 0.005) and the dorsolateral column had more non-reward correlated neurons (*χ*^2^_(2)_ = 13.86; *p* = 0.001) than expected based on the responses of all neurons recorded (Figure 6B). These data suggest that there may be a functional subdivision of reward responsiveness by column in the PAG, as there are with other functions such as defensive behavior. Importantly, not all PAG neurons within a column responded the same as there was still heterogeneity of responses within each column (Figures 6C–F).

## Discussion

While a vast literature supports the view that PAG mediates the processing of fear and pain information (Depaulis and Bandler, 2012), the present study provides the first clear evidence that PAG is also important for processing appetitive information, such as palatable rewards. We found that temporary and reversible inactivation of the PAG resulted in reduced reward consumption, decreased preference for large rewards, and more errors on a working memory task. An additional inactivation study involving a separate group of animals showed that the PAG is needed for normal food consumption in the absence of working memory demands, as evidenced by decreased consumption of as well as increased latency to consume food. This result was not simply due to motor deficits as general locomotion was generally increased. Finally, we recorded PAG neurons in awake behaving animals as they performed a working memory task to collect differential sized rewards. We found bidirectional encoding of reward encounters in a subset of PAG neurons, with some neurons exhibiting significantly increased firing rates during reward encounters while the other subset exhibiting significantly decreased firing.

Anatomical evidence supports the view that the PAG encodes information that is relevant to basic behaviors of the animal, such as sexual (Lonstein and Stern, 1998), pain and threat-avoiding (Rizvi et al., 1991; Krout et al., 1998), as well as food-seeking and consumption (Behbehani et al., 1988; Omelchenko and Sesack, 2010; Ntamati et al., 2018). Further, the Motivation-Decision model of pain (Fields, 2006), which states the decision to suppress responses to noxious stimuli under particular circumstances such as fear or an anticipated reward can engage descending modulatory pain circuits, would predict that the PAG may encode reward-relevant information when weighing behaviorally relevant stimuli to select the most appropriate action for a given situation. Indeed, we know that other brain regions canonically thought to be involved in defensive behaviors and threat detection, such as the amygdala and PBN, are also important for processing information relating to reward and food consumption. For example, the amygdala is important for reward and taste perception (Giraudo et al., 1998; Fontanini et al., 2009) and the PBN is essential for sensing gustatory stimuli and signaling satiety (Nicklous and Simansky, 2003; Scott and Small, 2009; Campos et al., 2016). Furthermore, both amygdala and PBN have reciprocal connections with the PAG, traditionally thought to be an important circuit for threat detection, anxiety, and fear (Rizvi et al., 1991; Krukoff et al., 1993; Krout et al., 1998). Together, these circuits may function to integrate other forms of salient information for adaptive action selection.

While the PAG projects to and receives projections from neurons in the VTA and amygdala, regions that show reward value prediction (Gottfried et al., 2003; Tobler et al., 2005), our current study does not support a role for PAG neurons in the encoding information that predicts reward value. PAG neurons’ reward responses did not show representations of expectation of reward size, as revealed by the reward switch and omission data. This is in contrast to other valuation systems in the brain, such as dopamine neurons, which show increased firing in response to rewards that are larger than expected as well as the inverse for smaller than expected rewards. Like the PPTg (Norton et al., 2011), another region that provides substantial excitatory input to VTA dopamine neurons, the PAG does not appear to be essential for determining prediction error codes.

A substantial subset (38%) of recorded PAG neurons are correlated with movement. It has been shown previously that many midbrain and hindbrain nuclei encode movement of the animal, i.e., PPTg, LDTg and VTA (Puryear et al., 2010; Norton et al., 2011; Redila et al., 2015). However, temporary inactivation of PAG did not cause motor impairment. Therefore, similar to suggestions made to account for strong velocity coding by VTA, LDTg, and PPTg neurons, PAG movement correlates may serve to keep track of ongoing behaviors to better prepare for future reward encounters. Indeed, in humans, it has been found that PAG neural activity increases when a threat is imminent, possibly in preparation for eliciting a predetermined flight motor pattern (Mobbs et al., 2007).

The food intake tests revealed that temporary inactivation of the PAG was sufficient to reduce food consumption in hungry animals. The PAG is an integral part of the endogenous opioid-mediated analgesia system and it is well known that opioids are important regulators of some aspects of food intake (Glass et al., 1999; Peciña and Berridge, 2000; Hagan et al., 2001; Will et al., 2003; Bodnar, 2004; Levine and Billington, 2004; Bodnar et al., 2005; Parker et al., 2014). The PAG may serve as an interface between the endogenous opioid system and hedonic aspects of food rewards. Indeed, evidence shows that opioid-mediated pain circuits are activated during appetitive circumstances (Fields, 2004). For example, chronic sucrose intake potentiates opioid mediated analgesia in the rat (Kanarek et al., 2001) and naloxone administration reduces motivation to work for sucrose rewards (Cleary et al., 1996). Sucrose administration was sometimes used as an effective substitute for analgesia in infants undergoing minor but painful procedures (Bucher et al., 1995; Stevens et al., 2004). While opioid signaling in the PAG negatively regulates food intake (Jenck et al., 1987), the PAG may play an important role in regulating the hedonic value of food signaled elsewhere. The reinforcing effects of systemic exogenous opiates such as heroin can be suppressed by intra-PAG infusions of naltrexone (Corrigall and Vaccarino, 1988) and infusion of morphine directly into the PAG is sufficient to induce conditioned place preference (Olmstead and Franklin, 1997; Le Merrer et al., 2009) suggesting that the PAG may be a necessary part of the opioid reinforcement circuit. Reward-associate cues also engage pain relief circuits. For example, a study with rats found that their withdrawal threshold to a painful stimulus was increased in an environment where they previously received a palatable food reward, an effect that was reversed with systemic administration of naloxone (Dum and Herz, 1984). Perhaps the reward responses the PAG neurons displayed in the current study are not responding to rewarding stimuli *per se*, but instead the neurons are activated in order to engage the descending pain modulatory circuit to favor approach behaviors to appetitive stimuli, especially in such cases that an animal is experiencing minor noxious stimuli. Thus, it is not surprising that inactivation of the PAG on the two tasks described here led to diminished food intake and increased latency to consume rewards as the circuit favoring ingestive behaviors was compromised. Additionally, compromising the descending pain-modulatory circuit may be experienced as unpleasant to the animal, which could further contribute to reduced food intake and lead to anxious behaviors such as increased locomotion.

Previous research has shown that the different columns of the PAG can be functionally divided because of their distinctly different contributions to autonomic processing, analgesia, and defensive behaviors (Bandler and Shipley, 1994). For example, injections of excitatory amino acids (EAAs) within the lateral and ventrolateral PAG column emit fundamentally opposite alterations in sensory responsiveness, and somatic and autonomic adjustments (Bandler and Shipley, 1994). The columns of the PAG can also be anatomically divided based on distinct projection populations and neurotransmitter expression (Carrive and Bandler, 1991; Onstott et al., 1993; Carrive and Morgan, 2012). The dorsolateral and lateral columns of the PAG contributed the majority of data in the current study. Between the two, we found that reward processing was functionally divided by column. We found that proportionally more lateral PAG neurons displayed excitation in response to reward encounters whereas proportionally more dorsolateral PAG neurons were not correlated to reward at all. This aligns with previous data that the lateral column of the PAG specifically is involved in food-oriented behaviors such as prey-hunting (Comoli et al., 2003; Mota-Ortiz et al., 2012). Prey-hunting behaviors mediated by the lateral column of the PAG are directly modified by opioidergic transmission (Miranda-Paiva et al., 2003; Sukikara et al., 2006). A conclusion that the reward-correlates are indeed a reflection of the opioid-mediated endogenous pain relief system becoming engaged is consistent with findings that the dorsolateral column of the PAG is not involved in opioid-mediated analgesia (Morgan, 1991).

We present data that provides novel evidence that the PAG may play a larger role in appetitive behaviors than previously shown. However, a limitation of the current study is that the pharmacological inactivation of PAG neurons was not selective to any particular neuron type or projection subpopulation. Additionally, the recording study does not tell us what type of neurons responded to reward and which did not. The most caudal regions of the PAG were not thoroughly examined, so the results reported here may not present the same from the caudal-most regions of the PAG. Nevertheless, complementing the predominantly separate literatures that document a role for the PAG in analgesia and defensive behaviors, the current study shows that the PAG is also an important region that integrates rewarding and ingestive behavioral information. This previously unstudied role should be further examined to gain insight into how neural systems interact to select the most adaptive behavior when competing motivations and stimuli are present. Understanding how neural systems that mediate threat detection and reward processing may interact and integrate for adaptive behavior selection will aide in understanding what is occurring in the brain when these systems have gone awry, such as in cases on anhedonia or anxiety disorders (Dillon et al., 2014).

## Author Contributions

VT and SM designed the study; wrote the manuscript. VT acquired the data; analyzed the data.

## Conflict of Interest Statement

The authors declare that the research was conducted in the absence of any commercial or financial relationships that could be construed as a potential conflict of interest.

## Supplementary Material

FIGURE S1 Left: reconstruction of bilateral cannulae tip placement (*N* = 7) that targeted the PAG in each individual rat. Numbers represent rat identification number. Colors represent different columns of the PAG. Blue = dorsolateral column; Green = lateral column; Purple = ventrolateral column. Right: results of Food Intake test and Latency to Consume Sucrose Test from Figure 2 with individual rat numbers.
