# Supplementary figures and images for: A Novel Role for the Periaqueductal Gray in Consummatory Behavior
Source: Front Behav Neurosci. 2018 Aug 28;12:178. doi: 10.3389/fnbeh.2018.00178 (PMC6121074; doi:10.3389/fnbeh.2018.00178)

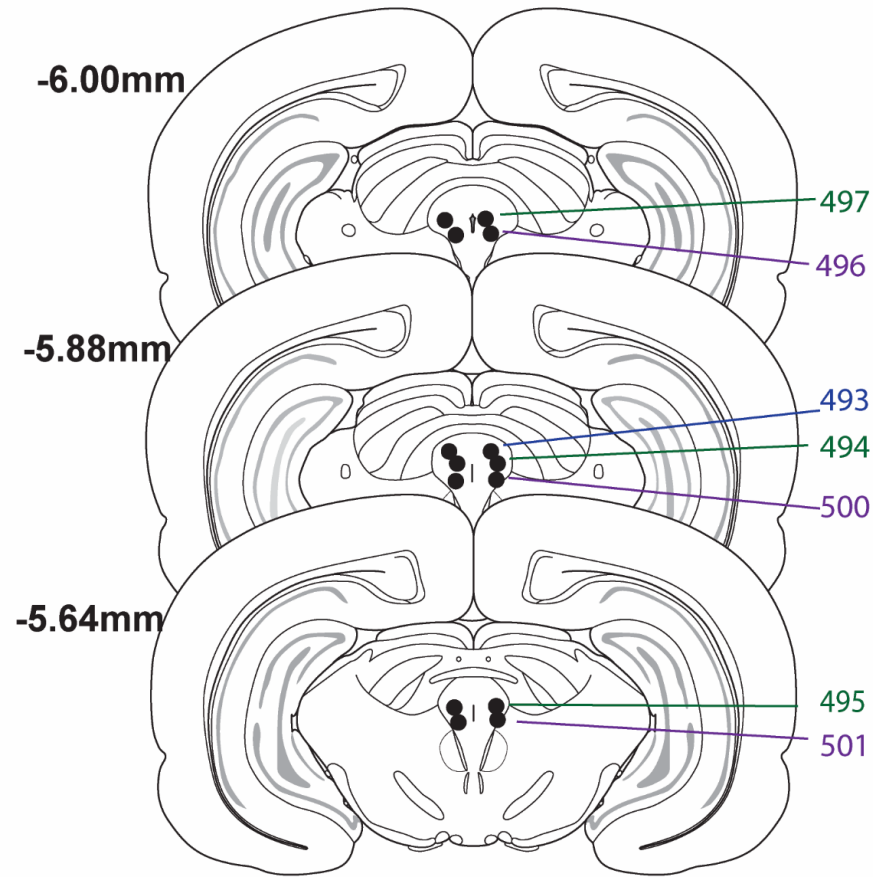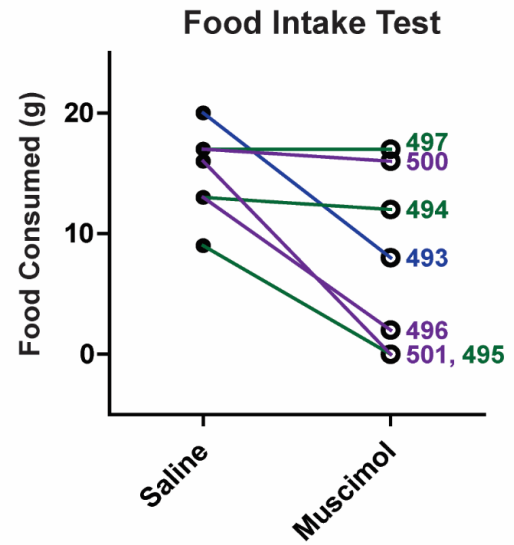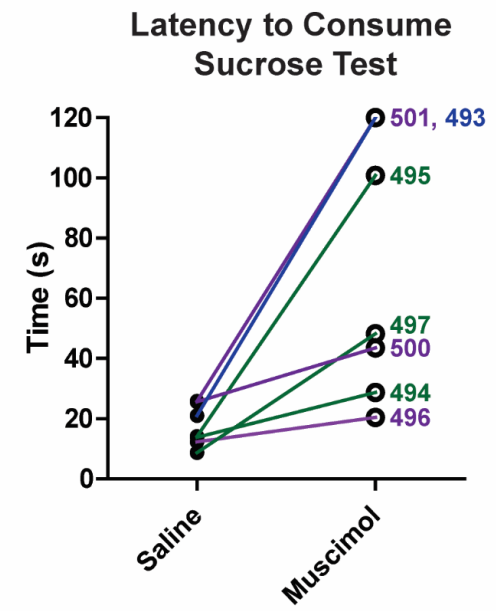

Supplement: FIGURE S1 — Left: reconstruction of bilateral cannulae tip placement (N = 7) that targeted the PAG in each individual rat. Numbers represent rat identification number. Colors represent different columns of the PAG. Blue = dorsolateral column; Green = lateral column; Purple = ventrolateral column. Right: results of Food Intake test and Latency to Consume Sucrose Test from Figure 2 with individual rat numbers. [file Data_Sheet_1.PDF]
